# Synthetic vs. Natural Hydroxytyrosol for Clean Label Lamb Burgers

**DOI:** 10.3390/antiox9090851

**Published:** 2020-09-10

**Authors:** Lorena Martínez-Zamora, Gaspar Ros, Gema Nieto

**Affiliations:** Department of Food Technology, Food Science and Nutrition, Faculty of Veterinary Sciences, Regional Campus of International Excellence “Campus Mare Nostrum”, Biomedical Research Institute of Murcia (IMIB-Arrixaca-UMU), University Clinical Hospital “Virgen de la Arrixaca”, University of Murcia, Espinardo, 30100 Murcia, Spain; lorena.martinez23@um.es (L.M.-Z.); gros@um.es (G.R.)

**Keywords:** antioxidant, hydroxytyrosol, lamb meat, burger, patties, volatile compounds

## Abstract

Clean labelling refers to consumers’ desire for manufacturers to be more transparent in the way their products are made and sourced. Natural antioxidants (spices, herbs, fruits, or vegetables) have been proven to offer the same functionality as their synthetic counterparts, with the advantage of being label friendly and process compatible, maintaining meat quality and reducing food waste. Lamb meat has the challenges to have an intense flavour and fat composition to test the effectiveness of some of these natural antioxidants like hydroxytyrosol (HXT). The current paper was designed to test both natural (HXTo) and synthetic (HXTs) antioxidants using four lamb patty batches: one Control (C) (which included sulphites); a reference (R) sample (14.6% carnosic acid and 6% carnosol from natural rosemary extracts, 200 ppm); a sample containing synthetic hydroxytyrosol (HXTs, 99% purity, 200 ppm); and a sample with added organic hydroxytyrosol (HXTo, sample 7% purity from olive tree leaves, 200 ppm). A shelf-life study was carried out for 6 days at 4 °C, testing proximal composition and mineral bioavailability, pH changes, colour (by CIELab), total antioxidant capacity (TAC by oxygen radical absorbance capacity (ORAC)), lipid and protein oxidation (thiobarbituric acid reactive substances (TBARs) and thiol loss, respectively), volatile compound profiles (by HPC-MS), sensory evaluation, and microbiological growth (as total vial count (TVC) and total coliform count (TCC)). Results revealed that lamb burgers with added HXTs had better-preserved raw lamb meat in the test conditions, with reduced colour losses, lipid oxidation, and release of volatile compounds, the half the microbiological growth (TVC) of the Control, the best TAC, and significantly increased (*p* < 0.05) minerals bioavailability, while maintaining sensory acceptability. In summary, natural antioxidants are an adequate strategy for lamb meat burgers. Regarding HXTo, obtained from olives, the synthetic analogue is even more effective in terms of preservative and antioxidant activity, and in maintaining the nutritional value, sensory characteristics, and safety of food products.

## 1. Introduction

Sheep were among the first livestock to be domesticated and used for their milk, wool, and meat. As early as the Neolithic age, from 9000 BCE, lambs were being raised in many parts of the Mediterranean and Central Asia. It is relatively easy to find high-quality lamb meat throughout the Mediterranean region, but it is mainly abundant in mountainous areas where lambs are raised in freedom. Lamb meat is considered to be a delicacy in the countries of the Mediterranean basin [1,2]. Lamb meat is a perishable product with a shelf-life of 7 to 10 days [1], because its high water activity (0.98) and pH (may reach pH 6) allow the growth of foodborne microbial pathogens [3]. For these reasons, some preservatives are used to ensure a better shelf-life. In the food industry, different types of antioxidant substances are added to try to delay or prevent the degradation of food. These substances can be of natural origin, such as ascorbate, citrate, and vitamin C; or of artificial origin, such as butylhydroxyanisole (BHA), butylhydroxytoluene (BHT), propyl gallate (PG), and butylhydroquinone (TBHQ). However, there is evidence that some of these compounds may be related to certain adverse effects, such as tumour development or allergic symptoms [4,5]. For this reason, there is a wide range of laws and regulations that strictly regulate the presence of these antioxidants in foods. In addition, current research and projects in this area are focused on replacing synthetic antioxidant additives with other compounds of natural origin, with similar preservative actions to these synthetic antioxidants in addition to providing potential health benefits.

One of the most important natural antioxidants is 3,4-dihydroxyphenylethanol or hydroxytyrosol (HXT). It is a highly bioactive alcoholic ortho-diphenol, which has been shown in several studies [6,7,8] to have interesting antioxidant and antimicrobial characteristics and to have beneficial effects on the cardiovascular system and on several human diseases. The list of biological activities is inexhaustible, including a negative regulation of the immune response, which protects human erythrocytes from hydrogen-peroxide-induced oxidative damage; as well as anti-inflammatory, antithrombotic, and hypocholesterolemic activities. It is also a potent monoamine oxidase (MAO-B) inhibitor, making it an ideal compound for the treatment of Alzheimer’s, Parkinson’s, and other neurological diseases [8,9]. It has also been shown to be a potent superoxide anion and hydroxyl radical scavenger. As a result of this broad antioxidant activity, HXT protects cells at different points in the human anatomy from damage and death, resulting in a lower frequency of cell death and a significant prolongation of the cell half-life [7].

Natural HXT does not exist in large quantities in its free form in nature, with the exception of olive trees (leaves, fruits, and waste waters from olive oil production), where it originates through the hydrolysis of oleuropein ((4*S*,5*E*,6*S*)-4-{2-[2-(3,4-dihydroxyphenyl)ethoxy]-2-oxoethyl}-5-ethylidene-6-{[(2*S*,3*R*,4*S*,5*S*,6*R*)-3,4,5-trihydroxy-6-(hydroxymethyl)-2-tetrahydropyranyl]oxy}-4*H*-pyran-3-carboxylic acid methyl ester). However, natural HXT extracts from olive trees contain a maximum of 7–25% pure HXT, together with other bioactive olive compounds. The natural mechanism by which the olive tree forms free HXT is enzymatic hydrolysis, involving the enzymes glycosidase and esterase [10]. In fact, several studies have considered procedures that generate high amounts of HXT through the use of by-products obtained after the milling and the extraction of olive oil. Wastewater from olive mills where olive oil is extracted has been studied, since such waters are very rich in free HXT [11]. Alternatively, synthetic enzymatic HXT procedures have been proposed [12]. However, the most used mechanism in the industrial processes to obtain this antioxidant is acid hydrolysis from oleuropein. In this way, HXT extracts can reach a purity of 94–99%. Similarly, rosemary has also been demonstrated to be a good protectant against lipid and protein oxidation in lamb meat products [13,14,15,16,17] and can be used as a reference model. For this reason, in this research rosemary extract has also been used as a control to compare the protective action of HXT.

Based on the above, the main objective of the present study was to conduct comparative tests of antioxidant extracts of HXT (from natural and synthetic sources) and rosemary to measure how these compounds affect the shelf-life of lamb meat burger patties. Thus, the antioxidant capacities of each extract were evaluated regarding their total phenolic contents.

## 2. Materials and Methods

### 2.1. Natural and Synthetic Antioxidants

Natural antioxidant extracts were supplied by Nutrafur-Frutarom, S.A. (Alcantarilla, Murcia, Spain): organic hydroxytyrosol (HXTo), obtained from vegetation waters from olive trees (*Olea europaea*), containing 7.26% pure bioactive compound; and rosemary extract (*Rosmarinus officinalis* L.) (R), containing 14.59% carnosic acid, 5.84% carnosol, and 0.60% 12-O-methylcarnosic acid. The synthetic hydroxytyrosol (HXTs) was obtained from the acid hydrolysis of oleuropein, containing 99.2% HXT and 0.3% HXT acetate, and was supplied by Seprox Biotech, S.L. (Fuente Álamo, Murcia, Spain).

### 2.2. Total Phenolic Content (TPC) and Antioxidant Capacity

For the development of these assays, HXT and rosemary extracts were diluted with Milli-Q water in a 1000 ppm solution.

The total phenolic content (TPC) was determined quantitatively following the method described by Singleton and Rossi [18], using the Folin–Ciocalteu reagent and gallic acid as the standard. Analyses were carried out in triplicate and the TPC was expressed as mg gallic acid equivalents (GAEs) per g of extract.

The antioxidant activity related to the chelating capacity was measured using the 2,2-diphenyl-1-picrylhydrazyl (DPPH) free radical scavenging method described by Brand-Williams, Cuvelier, and Berset [19]. Analyses were carried out in triplicate. The chelating activity percent was calculated applying this formula: ((Abs DPPH (control)—Abs DPPH (sample))/Abs DPPH (control)) × 100%.

The radical cation scavenging capacity against 2,2-azinobis (3-ethylbenzothiazolin)-6-sulphonic acid (ABTS) radical scavenging was carried out following the method described by Re et al. [20]. Then, 7 mM ABTS in 2.45 mM potassium persulphate (1:1, *v*/*v*), pH = 7.4, was adjusted to an absorbance of 0.7000 at 734 nm and mixed with 100 µL of sample. After 2 min of incubation, the absorbance was measured at 734 nm using a UV2 spectrophotometer (Pye Unicam Ltd., Cambridge, UK). Analyses were carried out in triplicate. The chelating activity percent was calculated using this formula: ((Abs ABTS (control)—Abs ABTS (sample))/Abs ABTS (control)) × 100%.

The ferric ion reducing antioxidant power assay (FRAP) was also performed following the method described by Benzie [21]. The FRAP reagent was prepared daily with a solution of 300 mmol/L acetate buffer, pH = 3.6, 20 mmol/L FeCl_3_·6 H_2_O, and 10 mmol/L TPTZ (2,4,6-tripyridyl-s-triazine) in 40 mmol/L HCl (10:1:1, *v*/*v*/*v*). After that, the FRAP reagent was mixed with 100 µL of sample in plastic cuvettes. The same procedure was carried out with a standard solution of Trolox. The absorbance was measured at 593 nm after 4 min incubation using a UV2 spectrophotometer (Pye Unicam Ltd., Cambridge, UK). Analyses were carried out in triplicate and the antioxidant capacity was expressed as µM Trolox equivalents (TEs) per g extract.

The oxygen radical absorbance capacity (ORAC) method [22] was followed to measure the hydrophilic antioxidant capacity. All dilution samples were prepared in triplicate. The antioxidant activity of the sample was expressed as µM of Trolox equivalents (TEs) per g of extract, using the following formula: (A × DF)/m, where A is the area under the curve of fluorescein, DF is the dilution factor, and *m* is the weight of the product.

### 2.3. Lamb Burger Preparation

Boneless leg of Segureño lamb was purchased in a local supermarket (Hipercor S.A., Murcia, Spain), brought to the laboratory within 30 min, and minced in an electric mincer (200 ppm) (Bosch MCM4, Gerlingen, Germany) for 4 min on the same morning they were bought, mixing all ingredients based on the recipes and ingredients proportions (Table 1). All patties were 80 g, and were handmade using sterile nitrile gloves for each batch. Samples were stored under aerobic conditions at 4 °C until analysis at days 0, 1, 3, and 6. Every batch was made of 60 patties (total 240), testing 15 lamb burgers per day of analysis: 3 for chemical analyses, 3 for microbiological analyses, and 9 for sensory analyses. Chemical and microbiological analyses was carried out in triplicate.

### 2.4. Proximal Composition and Mineral Bioavailability

Lamb burger samples were analysed for their moisture, ash, lipid, and total protein contents according to AOAC methods [23], on the same day as elaboration. The mineral concentrations of lamb burger samples were measured by plasma spectroscopy (ICP-OES) using an ICAP THERMO DUO 6500 computer after in vitro digestion [24] of the samples and expressed as bioavailable mineral fraction. The digestion procedure was divided into two phases performed at 37 °C: gastric and intestinal digestion. After digestion, samples were filtered (0.2 μm) and centrifuged at 223,487 *g* at 4 °C for 95 min (L-100XP optimal Ultracentrifuge Beckman Coulter, rotor 70Ti).

### 2.5. Shelf-Life Study

pH was measured at days 0, 1, 3, and 6 in triplicate (ISO 2917:1999) using Crison GLP21 equipment (Crison Instruments S.A., Barcelona, Spain).

Colour was measured using a Konika Minolta CR 400 chromameter (Minolta Camera Co., Osaka, Japan) standardised using a white calibration plate in triplicate. Lightness (L*), a* chromaticity (green–red), and b* chromaticity (blue–yellow) were measured according to the CIELab system. The colour coordinates were analysed at days 0, 1, 3, and 6 in triplicate.

Protein oxidation was measured in relation to the thiol concentration. The concentration of the thiol groups was determined after derivatisation by Ellman’s reagent, 5,5-dithiobis (2-nitrobenzoic acid) (DTNB) [25], following the method described by Martínez, Ros, and Nieto (2020) [26]. The absorbance was spectrophotometrically measured at 412 nm. The analyses were carried out at days 0, 1, 3, and 6 in triplicate.

Lipid oxidation was related to thiobarbituric acid reactive substances (TBARs) content, which was measured following the method described by Wang and Xiong [27]. The TBARs value (mg MDA/kg) was calculated using the formula (Abs_532_/m) × 9.48, where Abs_532_ was the absorbance obtained for each sample, m was the weight of the sample (g), and 9.48 a constant derived from the dilution factor and the molar extinction coefficient of the red TBA reaction product. The analyses were repeated in triplicate at days 0, 1, 3, and 6 of refrigerated (4 °C) storage.

#### 2.5.1. Microbiological Analysis

Microbiological growth of total vial count (TVC), total coliform count (TCC), and *Escherichia coli* was determined at days 0, 1, 3, and 6 from elaboration. Mass seeding was performed on PCA (TVC) and Rapid *E. coli* (to determine TCC and *E. coli*). Peptone water was used to make the dilutions. Analyses were performed in a laminar flow hood (Telstar, BIO-II-A, Madrid, Spain). After seeding, plates were incubated for 48 h at 37 °C for TVC, 24 h at 37 °C for TCC, and 48 h at 45 °C for *E. coli*. Analyses were performed in triplicate and expressed in cfu/g.

#### 2.5.2. Volatile Compounds by GC-MC

Lipid oxidation was also related to the concentration of volatile compounds and was determined following the method described by Martínez et al. [28]. Five grams of samples were placed in glass vials. Analyses were performed on a Hewlett-Packard 6890 N Series GC gas chromatograph fitted with a HP 5973 mass spectrometer and MSD Chemstation (Hewlett-Packard, Palo Alto, CA, USA). Compounds were identified by comparing their mass spectra with those contained in the NIST05 (National Institute of Standards and Technology, Gaithersburg, MD, USA) library. Volatile compounds were analysed in triplicate in all samples at days 0 and 6 from elaboration. Results were expressed in AU.

### 2.6. Sensory Analysis

A sensory analysis was carried out to measure the influence of incorporating natural extracts on the sensory properties of the meat products.

Ten panellists were previously trained following the ISO guide (2012). The tasting room was free of disturbing factors and air-conditioned.

Samples were coded with three random digits and were presented individually to the panellists. Sensory attributes were evaluated using an intensity scale from 1 (minimum: Undetectable) to 5 (maximum score: Very intense). Mineral water and stick breads were provided for mouth rinsing and palate cleansing between samples. The attributes measured for the colour, odour, and taste characteristics were: “Own Colour” (like lamb meat), “Extract Colour” (caused by added extracts), “Own Odour”, “Extract Odour”, “Own Flavour”, and “Extract Flavour”. A panel of twenty trained panellists measured the acceptability of lamb burgers. Sensory analysis was carried out on the same day as elaboration (day 0).

### 2.7. Statistical Analysis

Data were analysed with SPSS 23.0 (Statistical Package for the Social Science for Windows (IBM, Armonk, New York, NY, USA)). The results obtained from in vitro antioxidant capacity and shelf-life study were analysed using one-way ANOVA using “sample” and “time of analysis” as independent variables. A *p*-value < 0.05 was considered statistically significant. The Scheffe test was applied to test differences between groups.

## 3. Results

### 3.1. Characterisation of Preservative Extracts

The knowledge of the total phenolic content and the antioxidant activity of antioxidant ingredients allow a comparative evaluation between the preservative extracts analysed. The results obtained from each method are shown in Table 2.

The synthetic source of HXT presented the highest phenolic content, followed by the organic HXT and rosemary extract. The same behaviour was observed in the assays of total antioxidant and chelating capacities. Thus, the total phenolic content of HXTs was 140% higher than that in HXTo and R. Because of that, the oxygen radical absorbance capacity of HXTs was 7% higher than HXTo and 265% higher than the R extract, while the ferric-reducing antioxidant power of HXTs was 72% higher than HXTo and four times higher than R. Moreover, the chelating activity percent of HXTs against ABTS and DPPH radicals was only 15% and 9%, respectively, regarding the other two preservative extracts from *Olea europaea* and *Rosmarinus officinalis.* In these assays, all extracts presented a high scavenging potential for ABTS and DPPH radicals (>80%).

### 3.2. Proximate Composition and Bioavailability of the Mineral Fraction

As shown in Table 3, there were no significant differences between different reformulated samples and Control sample regarding the nutritional content. However, the bioavailable mineral fraction after in vitro digestion was significantly affected after adding phenolic extracts, especially with the incorporation of HXT. Only Fe and Si were affected by the presence of phenolic compounds. However, there were no significant changes in the concentration of the remaining minerals (Al, Ca, Cu, K, Li, Mn, Na, P, Rb, S, Sr, Zn, and Se) in prepared samples.

Lamb burger samples presented approximately 72% moisture, 1.8% ash, 14.5% protein, and 20.5% fat, with no significant differences. In addition, the concentration of Fe in samples with incorporated organic and synthetic HXT (HXTo and HXTs) was 54% and 63% higher than in the Control sample, respectively, while the sample prepared with rosemary extract (R) increased the bioavailable mineral fraction of Fe by 25% compared to the Control.

Furthermore, samples with incorporated HXTs and HXTo had concentrations of Si that were respectively increased by 185% and 165% compared to the Control. In R samples, the concentration of this mineral was increased by 108% with respect to the Control sample.

### 3.3. Shelf-Life Study

Table 4 summarizes the trends in the selected shelf-life indicators (pH, colour, and lipid and protein oxidation) during storage. As a common observation, all indicators declined with the time and for most of them the Control sample gave the highest values. pH decreased over the 6 days of storage in all samples, with HXTo samples having the most intense decline between days 0 and 1 and maintaining pH close to 5 throughout the storage period. HXT (both synthetic and organic) and R samples showed a very similar behaviour, while the Control sample (formulated with the Commercial mix^®^) maintained a pH closer to 6.

For colour parameters, lightness (L*) was maintained during the shelf-life study and there were no significant differences among different samples and days of analysis. However, redness tones (a*) of lamb patties decreased during the refrigerated storage. These changes were noticeable after the second day from elaboration (*p* < 0.05), when values of 20 units in the Control sample dropped to 14 and 11 units by days 3 and 6. Thus, the decline was more pronounced in Control, since samples reformulated with HXT and R attenuated this decrease by 33% compared to the commercial sample (Control). Similarly, yellowness tones (b*) of samples were also affected by the incorporation of natural extracts to lamb patties. A reduction of b* parameters can be observed in Table 4 (*p* < 0.05), but this decrease was more gradual in the case of samples made with HXT and rosemary extracts. In fact, the b* of reformulated samples decreased by one or two units, while the b* values of Control samples decreased by six units, 75% more.

Regarding the lipid oxidation, the sample that reported the lowest TBARs value was HXTs, followed by Control, HXTo, and R, which gave the same value of oxidation after six days of refrigerated storage (*p* < 0.05). This demonstrates how the incorporation of synthetic HXT with 99% purity (HXTs) reduced the oxidation of lamb patties by 35% with respect to the Control sample, which incorporated sulphites and synthetic preservatives. In contrast, the sulphites and synthetic antioxidants of the Control sample reduced the thiol loss by 50% compared to the rest of the reformulated samples (HXTo, HXTs, and R) from the beginning to the end of the shelf-life study under refrigerated storage. However, a reduction of the thiol concentration by approximately 85% can be appreciated through the preservation time under refrigerated conditions.

Regarding the microbiological content of lamb patties, results of total vial count, total coliform count, and *E. coli* growth are shown in Table 5.

As can be seen, the Control sample presented the highest concentration of TVC followed by HXTo, R, and HXTs, which demonstrated that the synthetic source of HXT (99% purity) inhibited almost twice the total microbiological growth with respect to the commercial mix made with synthetic additives. Therefore, the antimicrobial capacity of HXTs was higher than the combination of sulphites, sodium ascorbate, and sodium citrate (Commercial mix^®^), even though the TCC was 50% inhibited by this formula regarding HXTs and HXTo.

### 3.4. Volatile Compounds (GS-MS)

The results obtained from the analysis of the volatile compounds released by the different samples showed that there were numerous and diverse species volatilised during preservation. Only the compounds that presented repeatability and significance have been selected and shown in Table 6. The values obtained as chromatographic peaks are shown as the volatile compound profiles of the studied samples.

All volatile compounds significantly increased (*p* < 0.05) from day 0 to day 6 under refrigerated storage.

Certain compounds from amino acid catabolism, such as 2,3-butanediol, 3-methyl-1-butanal, or 3-methyl-1-butanol, were identified, which is a good indicator of meat degradation. As can be seen, the control sample presented the highest values, while samples enriched in HXT (both organic and synthetic) reported the lowest values of these compounds released from protein degradation.

Other volatile compounds derived from lipid oxidation (e.g., hexanal, nonanal, and 2-bornanone) were also identified. In this case, the control sample also showed the highest values in comparison to HXTs and HXTo, which presented values between three and ten times lower than Control.

### 3.5. Sensory Analysis

**Figure 1:**
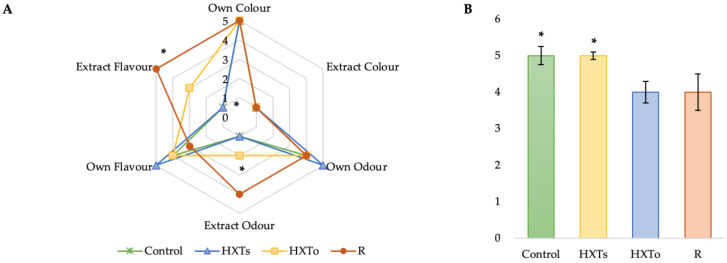
Sensory analysis (**A**) and Acceptability (**B**) of lamb patty samples. HXTs: samples enriched in synthetic hydroxytyrosol, HXTo: samples enriched in organic hydroxytyrosol; R: samples enriched in rosemary. *: Indicates significant differences between samples (*p* < 0.05).

Figure 1 represents the sensory properties of the studied samples, as well as the general acceptability measured by a consumer panel.

As can be seen in Figure 1A, the R sample presented the highest values of “Extract Flavour” and “Extract Odour” (*p* < 0.05), while the rest of samples did not present significant differences with respect to appreciable colour, odour, or flavour. Similarly, Control and HXTs samples did not present significant differences between each other regarding acceptability; they obtained the highest score (Figure 1B), and these results were significantly different from those for HXTo and R (*p* < 0.05).

## 4. Discussion

Regarding the characterisation of the natural extracts used, the synthetic source of HXT presented the highest phenolic content, as well as the highest antioxidant capacity as measured by several methods (ORAC, FRAP, DPPH, and ABTS), as seen in Table 2. This was due to the purity of HXTs (99.2%) compared to the 7.26% content of bioactive compound in HXTo. HXT is known for its antioxidant potential, which is ten times higher than green tea and two times greater than coenzyme Q10 [29]. It also has a greater function as a free radical scavenger, and greater efficacy under stress conditions. This antioxidant activity is based on the chemical structure of this phytochemical compound: a phenol ring formed by a catechol group and three hydroxyl groups. The combination of these functional groups in this molecule is the main reason for its preservative action [7] in animal products, as our research group has previously demonstrated [11,28,30,31,32].

Similarly, rosemary has also been demonstrated to have important antimicrobial and antioxidant qualities due to its phenolic acid and flavonoid content. Carnosol and carnosolic acid have been identified as among the most effective antioxidant constituents of rosemary. In addition, its extract contains carnosic acid, epirosmanol, rosmanol, methylcarnosate, and isorosmanol [14,26,33,34]. Furthermore, these extracts have also previously demonstrated antioxidant properties, both in vitro and applied to meat products [29,30,35].

Once natural extracts were characterised in vitro, they demonstrated their bioactivity as preservatives in lamb patties.

First, the prepared clean label lamb patties did not report any difference (*p* < 0.05) with respect to proximate composition; however, samples with HXT incorporated into their formula showed an increased bioavailability of minerals Fe and Si (Table 3). Thus, HXT is a compound linked to minerals, as has been reported with gluconate Fe (II) in black olives, which catalyses the oxidation of this compound, so it is possible that HXT influences the biological bioavailability of some minerals and trace elements [34]. This behaviour was also shown by Martínez, Ros, and Nieto [35], when the presence of HXT in chicken meat emulsions increased the bioavailability of Fe in a Caco-2 cell model. In the present study, the same occurred with Si, but there is no previous research that can justify this increase in the bioavailability. A possible theory may be an affinity between HXT and Si, since they have been studied together as antioxidants in restructured pork that contribute to beneficial health effects, such as the amelioration of metabolic syndrome or liver oxidation status [36,37].

Secondly, the shelf-life study indicated that HXTs and HXTo can be used as substitutes for sulphites and synthetic antioxidants in fresh lamb patties.

The decrease of pH values is a normal occurrence in fresh meat during its preservation, as other researchers have previously reported [1,2,38,39]. Moreover, the results obtained presented no significant differences between different treatments, hence the incorporation of HXT and R did not affect muscle pH, in agreement with previous studies that analysed the incorporation of natural extracts to lamb patties [40,41].

Regarding the colour evolution, there was a variation from the intense pinkish colour that is characteristic of fresh lamb meat towards browner shades with less luminosity, in which the Control sample was the most affected (Table 4). One of the main functions of antioxidants is the maintenance of colour, which is part of the antioxidative activity of the preservative agents HXT and R in this case. The mechanisms of action of this antioxidant effect includes the chelating effect of phenols (from HXT and R) on free iron ions from heme-group degradation, protecting them from the catalysis of lipid oxidation reactions. For that reason, these results agree with the first results of the antioxidant capacity (Table 1). HXTs presented the highest values of antioxidant power as measured by FRAP, which can justify the protection of this extract against colour losses and browning due to haemoglobin oxidation. This is consistent with previous studies in which these extracts were applied in meat [31,35] and lamb products [16,40,41,42,43,44].

In the same way, all the incorporated extracts better protected the lamb patties against lipid oxidation compared to the Control sample, until the sixth day of the refrigerated preservation, when only HXTs was able to protect the fresh meat against this phenomenon (Table 4). Lamb meat is particularly sensitive to the effects of lipid degradation due to the high quantity of polyunsaturated fatty acids of the muscle, which are more susceptible to oxidation phenomena. In fact, lamb meat is comprised of 20% fat (Table 3), from which 7% of the total weight are saturated fatty acids, while the rest (13%) is formed by monounsaturated and polyunsaturated fatty acids [44,45]. The lipid peroxidation in meat products is produced by a radical chain reaction mechanism, and this is accelerated by the presence of oxygen. In addition, lipid oxidation occurs due to several factors, such as free radicals, a deficit of antioxidants, polyunsaturated fatty acids concentration (PUFA), added salt, and a high concentration of prooxidants. At the same time, these reactions produce secondary products like reactive carbonyl species 4-HNE (4-hydroxynonenal) and MDA (malondialdehyde), reactive oxygen species (ROS) like superoxide anion and hydroxyl radical, perferryl and ferryl species, and lipid peroxyl radical. These compounds are responsible for the rancid flavour in animal products. Thus, the addition of antioxidants to lamb products is important in order to avoid the appearance of rancid flavours due to the release of malondialdehyde as a by-product of lipid oxidation reactions. Therefore, the great antioxidant activity of HXT (Table 2) [7,46] confirms what other authors have previously stated about HXT and its ability to protect lamb meat against oxidation [39,42,47], and other meat products such as chicken sausages [11,31] or chicken nuggets [26].

In contrast, the incorporation of HXT and R increased the thiol loss from the elaboration until the end of the shelf-life study by 50%, which was directly related to the protein oxidation of the raw meat (Table 4). The main reaction products of this oxidation are protein disulphides, which have been demonstrated to have a strong impact on the quality of meat, which can be appreciated as an increase of the toughness and a decrease of the tenderness of the meat. Furthermore, the oxidation of myofibrillar proteins by hydroxyl radicals (OH^•^) leads to cross-link formation and consequent disulphide formation, which is related to increased myosin heavy chain (MHC). The cross-linked MHC has been correlated with a significant decrease in tenderness by several authors in pork patties [48], and in raw pork meat [49,50]. The oxidation of the backbone of a protein promotes the modification in the atoms of the polypeptide chain, as well as fragmentation, aggregation, and polymerisation of the proteins. Myosin is the most affected protein, among other amino acids that are especially sensitive to ROS, such as arginine, cysteine, histidine, lysine, methionine, phenylalanine, proline, tryptophan, and tyrosine. Nevertheless, this prooxidant reaction after application of HXT and R can be explained by the multiple reaction sites on rosmarinic acid and HXT, including the o-catechol rings [51]. It is proposed that the thiol loss caused by the addition of phenolic compounds to meat products may result in the formation of covalent bonds between protein thiol groups and quinones as oxidised ο-catechol. Carnosic acid and carnosol (R), as well as HXT, each contain one possible site of reaction. Recently, we also showed the same behaviour when applying rosemary in an oxidised pork meat system, where a low radical signal intensity was observed by ESR due to phenoxyl radicals produced by quinones from rosmarinic acid [52]. This can explain the potential antioxidant activity of R and HXT and their prooxidant activity against thiol groups, which can also be extrapolated to the present study.

Again, HXTs also presented the highest antimicrobial activity against total vial count. Furthermore, the Control sample, which was made using sulphites and synthetic antioxidants, inhibited the growth of coliform bacteria to a greater extent (Table 5). However, no sample surpassed the legal limits established by the Spanish and European Law for this kind of product. The antimicrobial power of these natural extracts resides in the presence of phenolic compounds, which are biologically active with potential antimicrobial activity. These phytochemicals act by inhibiting cellular protein synthesis due to the formation of irreversible complexes with proteins. This makes it understandable that the biological properties of extracts rich in flavonoids or phenolic acids, like HXT and R, have an increased antimicrobial activity against bacterial growth. This behaviour can also be compared to previous research carried out by Tafesh et al. [53], which also demonstrated the antibacterial power of olive extracts (as sources of HXT) in lamb patties. Similarly, Bañón, Méndez, and Almela [13] also demonstrated the antimicrobial activity of rosemary after its endogenous application in lamb meat through its dietary administration, while Alkass, Baker, and Saleh [54] and Andrés et al. [40] proved the same activity after its exogenous application during lamb patty manufacturing.

Regarding the volatile compound profile, the incorporation of HXT and R reduced the release of compounds related to amino acid catabolism and lipid oxidation (Table 6). Lipolysis (due to lipid oxidation) and amino acid catabolism might be the most important pathways for the production of volatile compounds in meat. The production of 2,3-butanediol, 3-methyl-1-butanal, or 3-methyl-1-butanol from leucine, valine, and isoleucine is explained by amino acid degradation, and is a good indicator of protein oxidation. However, this cannot be related to the results obtained from thiol groups, because HXT and R seem to act as prooxidants by the reaction between o-catechol groups and thiol groups forming thiol-quinone adducts, as previously described.

In the same way, volatile compound profiles deriving from lipid oxidation (hexanal, nonanal, and 2-bornanone) (Table 6) were also affected by the addition of HXT and R (*p* < 0.05), which can be directly related to lower values of MDA (Table 4). Therefore, the release of hexanal and nonanal is also associated with the apparition of rancid flavours, as well as painty and waxy descriptors. For this reason, the decrease of the volatile compound profile in reformulated samples is another proof of the antioxidant and preservative capacity of the extracts used (HXTs, HXTo, and R), which improved the control of the lipid degradation due to their antioxidant ability (Table 2). These results also agree with the research of Alkass, Baker, and Saleh [54], who also showed the reduction of volatile compounds related to oxidative rancidity after application of rosemary (0.05%) and ginger (0.5%) extracts in Karadi lamb patties stored at −18 °C for 150 days. In a similar way, HXT has previously been tested in raw fish patties, in which a reduction of the volatile compounds related to lipid peroxidation and amino acid catabolism was observed [30].

The highest acceptability was obtained in the samples containing HXTs, at the same level as the Control sample (Figure 1B), while samples with added HXTo and R reported a decrease of this parameter because of the elevated “Extract Flavour” detected by the trained panellists (*p* < 0.05) (Figure 1A). This “Extract Flavour” was highly noticeable due to the organic origin of these extracts and the impurities from olive and rosemary that influence the taste of the lamb meat. Furthermore, the synthetic origin of HXTs leads to a cleaner extract, of an increased purity (99%), with no strange flavours that could alter the perception of the meat formulations.

## 5. Conclusions

HXT extracts showed a great antioxidant activity in vitro, followed by R. Both of the HXT extracts and the R extract showed a good preservative activity in most of the parameters studied—even higher than the Control sample made with sulphites and synthetic antioxidants. However, HXTs showed the best results: excellent protective effect against lipid oxidation and microbiological growth, with good colour maintenance and general acceptability. However, all the extracts acted as prooxidants against thiol groups, possibly due to the formation of thiol-quinone adducts between free thiol groups of the proteins and o-catechol groups of bioactive compounds from HXT and R. Therefore, HXTs samples reported the best physical-chemical, microbiological, and organoleptic quality.

## Figures and Tables

**Table 1:** Formulation of lamb burgers.

Ingredients	Control	HXTs	HXTo	R
Lamb meat (g)	2560	2560	2560	2560
Water (mL)	640	640	640	640
Commercial mix^®^ (g/kg)	69			
Preservative extracts (ppm)				
• HXTS		200		
• HXTO			200	
• R				200

Commercial mix^®^: spices, salt, dextrose, lactose, soy protein, emulsifiers (sodium phosphate E-451), preservative (sodium sulphite E-221), antioxidant (sodium ascorbate E-301 and sodium citrate E-331). HXTs: synthetic hydroxytyrosol (99% purity); HXTo: organic hydroxytyrosol (7% purity); R: rosemary (14.6% carnosic acid and 6% carnosol).

**Table 2:** Total phenolic content (mg GAE g^−1^), chelating activity percent (%), and antioxidant activity (µmol TE g^−1^) of preservative extracts tested.

Sample	Total Phenolic Content	Antioxidant Activity			
		ABTS	DPPH	ORAC	FRAP
**HXTs**	93.9 ± 54 ^a^	93.3 ± 5.3 ^a^	88.9 ± 3.9	70,542 ± 299.6 ^a^	64,961 ± 1239.4 ^a^
**HXTo**	41.6 ± 81 ^b^	82.2 ± 4.5 ^b^	81.9 ± 1.3	40,993 ± 285.7 ^b^	60,457 ± 1439.4 ^b^
**R**	36.5 ± 26 ^c^	80.1 ± 5.1 ^b^	81.3 ± 5.0	13,929 ± 393.4 ^c^	17,790 ± 839.4 ^c^

HXTs: synthetic hydroxytyrosol; HXTo: organic hydroxytyrosol; R: rosemary. a–c: Different letters within the same column indicate significant differences between samples (*p* < 0.05).

**Table 3:** Proximate composition and bioavailable mineral fraction of lamb burger samples.

	Proximate Composition (Average Percentage ± SD)				Mineral Bioavailability (mg/100 g)	
Samples	Moisture	Ash	Protein	Lipid	Fe	Si
**Control**	72.82 ± 0.26	2.01 ± 0.09	14.28 ± 0.81	19.88 ± 0.73	1.13 ± 0.00 ^c^	23.53 ± 0.03 ^d^
**HXTs**	72.60 ± 0.91	1.93 ± 0.15	14.00 ± 0.19	19.19 ± 1.07	1.85 ± 0.16 ^a^	67.19 ± 0.08 ^a^
**HXTo**	71.33 ± 1.20	1.72 ± 0.07	14.43 ± 0.58	20.37 ± 0.30	1.75 ± 0.04 ^a^	62.53 ± 0.10 ^b^
**R**	73.04 ± 2.49	1.70 ± 0.19	15.24 ± 0.19	22.42 ± 2.09	1.42 ± 0.01 ^b^	48.95 ± 0.05 ^c^

HXTs: samples enriched in synthetic hydroxytyrosol; HXTo: samples enriched in organic hydroxytyrosol; R: samples enriched in rosemary extract. The mineral concentrations of lamb burger samples after in vitro digestion are expressed as bioavailable mineral fraction. Only significant results are shown. a–d: Different letters within in the same column indicate significant differences between samples (*p* < 0.05).

**Table 4:** Physical-chemical quality evolution of lamb patties for six days of refrigerated storage.

Days of Storage				
Samples	0	1	3	6
**pH**				
**Control**	6.12 ± 0.04	5.86 ± 0.00	5.79 ± 0.01	5.76 ± 0.01
**HXTs**	6.10 ± 0.00	5.38 ± 0.01	5.35 ± 0.01	5.42 ± 0.01
**HXTo**	6.05 ± 0.01	5.06 ± 0.01	4.97 ± 0.01	5.07 ± 0.01
**R**	5.84 ± 0.00	5.30 ± 0.00	5.22 ± 0.00	5.31 ± 0.01
L*				
**Control**	55.36 ± 1.30	54.73 ± 1.61	53.75 ± 2.46	55.88 ± 2.47
**HXTs**	53.53 ± 1.74	55.57 ± 2.65	56.96 ± 1.53	56.27 ± 1.58
**HXTo**	54.36 ± 1.54	55.12 ± 1.02	57.11 ± 1.52	57.08 ± 0.63
**R**	52.63 ± 1.55	54.88 ± 1.01	56.18 ± 1.79	54.19 ± 1.16
a*				
**Control**	20.17 ± 0.27 ^a,x^	21.14 ± 0.51 ^a,x^	14.66 ± 1.03 ^a,y^	11.11 ± 0.27 ^z^
**HXTs**	17.89 ± 0.16 ^b,x^	16.33 ± 0.43 ^b,y^	11.81 ± 0.28 ^b,z^	11.52 ± 0.18 ^z^
**HXTo**	17.69 ± 0.30 ^b,x^	16.44 ± 0.42 ^b,y^	11.45 ± 1.31 ^b,z^	11.83 ± 0.47 ^z^
**R**	17.32 ± 0.50 ^b,x^	16.27 ± 1.37 ^b,x^	12.16 ± 0.16 ^b,y^	11.21 ± 0.41 ^z^
b*				
**Control**	14.73 ± 0.08 ^a,x^	14.61 ± 0.29 ^a,x^	10.67 ± 0.89 ^b,y^	8.52 ± 0.19 ^c,z^
**HXTs**	12.42 ± 0.24 ^b,x^	11.82 ± 0.13 ^c,y^	11.27 ± 0.21 ^b,y^	10.57 ± 0.16 ^b,z^
**HXTo**	12.38 ± 0.19 ^b^	11.57 ± 0.17 ^c^	12.05 ± 0.79 ^a^	11.25 ± 0.16 ^a^
**R**	12.41 ± 0.31 ^b,y^	12.16 ± 0.20 ^b,y^	12.07 ± 0.24 ^a,y^	10.26 ± 0.32 ^b,z^
*** Lipid Oxidation: TBARs (mg MDA/kg)***				
**Control**	1.36 ± 0.22 ^a,y^	1.71 ± 0.26 ^y^	1.37 ± 0.09 ^b,y^	1.00 ± 0.15 ^b,z^
**HXTs**	0.70 ± 0.10 ^b,z^	1.42 ± 0.25 ^x^	1.12 ± 0.12 ^c,y^	0.65 ± 0.06 ^c,z^
**HXTo**	0.84 ± 0.03 ^b,z^	1.62 ± 0.11 ^x^	1.56 ± 0.14 ^a,x^	1.19 ± 0.04 ^a,y^
**R**	0.78 ± 0.08 ^b,z^	1.44 ± 0.05 ^x^	1.15 ± 0.08 ^c,y^	1.13 ± 0.10 ^a,y^
** *Protein Oxidation: Thiol Loss (nmol/mg protein)***				
**Control**	29.04 ± 1.15 ^a,x^	21.78 ± 3.00 ^a,y^	6.48 ± 0.22 ^a,z^	5.75 ± 0.28 ^a,z^
**HXTs**	15.16 ± 1.26 ^b,x^	14.13 ± 1.87 ^b,x^	3.76 ± 0.17 ^b,y^	2.12 ± 0.06 ^b,z^
**HXTo**	18.69 ± 1.98 ^b,y^	16.26 ± 2.03 ^b,y^	2.54 ± 0.04 ^c,z^	2.25 ± 0.14 ^b,z^
**R**	17.54 ± 2.54 ^b,y^	16.33 ± 1.25 ^b,y^	2.54 ± 0.01 ^c,z^	2.01 ± 0.08 ^b,z^

HXTs: samples enriched in synthetic hydroxytyrosol; HXTo: samples enriched in organic hydroxytyrosol; R: samples enriched in rosemary. a–d: Different letters within the same column indicate significant differences between samples (*p* < 0.05). x–z: Different letters within in the same row indicate significant differences between samples at different times of analysis (*p* < 0.05).

**Table 5:** Evolution of microbiological content (cfu/g) of lamb patties for six days of refrigerated storage in aerobic conditions.

	Days of Storage				
Microorganism	Samples	0	1	3	6
TVC	**Control**	6350 ± 40 ^a,z^	25,000 ± 1200 ^c,y^	140,000 ± 25000 ^a,x^	220,000 ± 4250 ^a,w^
	**HXTs**	3600 ± 50 ^c,z^	53,000 ± 650 ^a,y^	59,000 ± 4000 ^c,x,y^	80,000 ± 1150 ^d,w^
	**HXTo**	1550 ± 46 ^d,z^	55,000 ± 1500 ^a,y^	80,000 ± 5000 ^b,x^	180,000 ± 8000 ^b,w^
	**R**	4750 ± 34 ^b,z^	40,000 ± 700 ^b,y^	50,000 ± 2500 ^d,x,y^	107,000 ± 9500 ^c,w^
TCC	**Control**	895 ± 70 ^b,z^	950 ± 80 ^c,y,z^	1280 ± 210 ^c,x,y^	2000 ± 120 ^c,w^
	**HXTs**	3490 ± 67 ^a,z^	3000 ± 90 ^a,z^	3400 ± 200 ^b,z^	4000 ± 320 ^b,y^
	**HXTo**	3280 ± 110 ^a^	3930 ± 50 ^c^	3800 ± 380 ^b^	4900 ± 860 ^b^
	**R**	515 ± 48 ^c, z^	1460 ± 115 ^b,y^	8000 ± 190 ^a,x^	9300 ± 250 ^a,w^
*E. coli*	<10				

HXTs: samples enriched in synthetic hydroxytyrosol; HXTo: samples enriched in organic hydroxytyrosol; R: samples enriched in rosemary. TVC: total vial count; TCC: total coliform count. a–c: Different letters within the same column indicate significant differences between samples (*p* < 0.05). w–z: Different letters within the same row indicate significant differences between samples at different time of analysis (*p* < 0.05).

**Table 6:** Evolution of the volatile compound profiles (a.u.) of lamb patties samples for six days of refrigerated storage in aerobic conditions (M ± SD).

	Sample	Day 0	Day 6		Day 0	Day 6
3-Methyl-1-butanol	**Control**	3.10 ± 0.32	6.60 ± 0.01 ^a^	3-Methyl-1-butanal	0.45 ± 0.07	1.78 ± 0.02 ^a^
	**HXTs**	0.22 ± 0.03	2.16 ± 0.03 ^b^		0.12 ± 0.00	0.44 ± 0.03 ^d^
	**HXTo**	1.01 ± 0.05	1.42 ± 0.05 ^c^		0.25 ± 0.02	0.60 ± 0.05 ^c^
	**R**	0.45 ± 0.01	2.92 ± 0.04 ^b^		0.66 ± 0.04	1.35 ± 0.12 ^b^
Hexanal	**Control**	0.03 ± 0.00	1.99 ± 0.02 ^b^	2-Bornanone	0.01 ± 0.00	0.38 ± 0.02
	**HXTs**	0.02 ± 0.05	0.63 ± 0.02 ^c^		nd	nd
	**HXTo**	0.03 ± 0.01	0.59 ± 0.05 ^c^		nd	nd
	**R**	0.03 ± 0.01	5.88 ± 0.03 ^a^		nd	nd
2,3-Butanediol	**Control**	0.16 ± 0.01	10.56 ± 0.02 ^a^	Nonanal	0.56 ± 0.00	1.70 ± 0.10 ^a^
	**HXTs**	0.29 ± 0.01	3.33 ± 0.03 ^d^		0.03 ± 0.00	0.10 ± 0.00 ^c^
	**HXTo**	1.09 ± 0.01	6.60 ± 0.05 ^c^		0.11 ± 0.02	0.31 ± 0.03 ^b^
	**R**	0.39 ± 0.01	7.35 ± 0.01 ^b^		0.06 ± 0.01	0.43 ± 0.01 ^b^

HXTs: samples enriched in synthetic hydroxytyrosol; HXTo: samples enriched in organic hydroxytyrosol; R: samples enriched in rosemary extract. a–c: Different letters among data in the same column indicate significant differences between samples (*p* < 0.05). nd: undetected values.
